# Anemia Prevalence and Risk Factors in Two of Ethiopia's Most Anemic Regions among Women: A Cross-Sectional Study

**DOI:** 10.1155/2023/2900483

**Published:** 2023-12-09

**Authors:** Gebru Gebremeskel Gebrerufael, Bsrat Tesfay Hagos

**Affiliations:** ^1^Department of Statistics, College of Natural and Computational Science, Adigrat University, Adigrat, Ethiopia; ^2^Department of Statistics, College of Natural and Computational Science, Mekelle University, Mekelle, Ethiopia

## Abstract

**Background:**

In Sub-Saharan African (SSA) nations, including Ethiopia, anemia is a significant public health issue. Ethiopia has continued to bear the enormous burden of anemia infections. Over time, the prevalence of anemia has significantly increased in Ethiopia. In addition, there is a paucity of literature and regional variations in the pace of increment expansion. Therefore, the primary goal of this study was to evaluate the prevalence of anemia and risk factors among women in Ethiopia's two most anemic regions.

**Methods:**

2,519 women participated in a community-based cross-sectional study from January 18 to June 27, 2016. In order to determine the causes of anemia in women in two of Ethiopia's most anemic regions, an ordinal logistic regression model was taken into consideration. The applicability of the proportional odds test was evaluated using the chi-square test of the parallelism assumption. A *p* value of 0.05 or below was used to define crucial and statistically significant predictor variables.

**Results:**

The overall prevalence rate of anemia was 56.8% (95% CI (54.8%–58.7%)). The chi-square test of the parallelism assumption indicated that the odds ratios were constant across all cut-off points of women's anemia levels at a 5% significance level (*p* value = 0.122). Of the severity of anemia levels among women, 48.2, 46.1, and 5.7% had mild, moderate, and severe anemia levels, respectively. In multivariable ordinal logistic regression analyses, being born (lived) in the Somali region (AOR = 1.6, 95% CI: 1.37, 1.90), having a parity of 4–5 (AOR = 1.3, 95% CI: 1.05, 1.66), and having ≥6 children (AOR = 1.4, 95% CI: 1.1, 1.7), being a contraceptive user (AOR = 3, 95% CI: 2.5, 3.6), being currently pregnant (AOR = 2.8, 95% CI: 2.3, 3.4), having no ANC follow-up (AOR = 1.9, 95% CI: 1.6, 2.3), being married women (AOR = 1.4, 95% CI: 1.1, 1.9), and user of unimproved toilet facility (AOR = 1.3, 95% CI: 1.1, 1.6) were significantly positively associated with anemia.

**Conclusions:**

Finally, the anemia burden was dangerously greater than the national average. The region, usage of contraceptives, being pregnant at the time, ANC follow-up, toilet facilities, parity, and marital status all had a substantial impact on anemia. Therefore, to lessen the prevalence of anemia in certain parts of Ethiopia, public health initiatives that improve maternal health service utilization are required, such as ANC follow-up to minimize parity.

## 1. Background

Low hemoglobin (Hgb) levels in the blood, which reduce red blood cells' ability to transport oxygen to tissues, are the hallmark of anemia [1–5]. Anemia is one of the primary public health issues linked to the 2 billion individuals worldwide who suffer from micronutrient-deficient conditions [2–4]. Women and children are more vulnerable to anemia despite the fact that it affects people at all stages of life; the prevalence rate for anemia worldwide is 47% in children under the age of 5, 42% in pregnant women, and 30% in nonpregnant women aged 15–49 [6]. Anemia causes 20% of maternal deaths worldwide and is linked to newborn mortality, preterm birth, and low birth weight [3, 7].

African nations with an endemic malaria parasite problem still struggle with anemia [8]. The prevalence rate of anemia among women in the reproductive age range (15–49 years) is 37.6% in the African area [9]. In addition, the SSA countries have the highest prevalence rate, with 57% of pregnant women suffering from anemia [6]. Anemia has persisted as a serious public health issue in Ethiopia. It has serious effects on both the mother and the fetus [3]. According to several risk variables, the prevalence of anemia sickness among women varied across the nation's regions [10, 11]. The occurrence of several contextual and geographic predictor variables, such as diet and the prevalence of communicable diseases, may be the cause of these discrepancies [12]. In addition, according to the 2016 EDHS data, anemia prevalence in Ethiopia is greater (said to be 24%), with the highest rates found in the regional states of Afar and Somalia, where they were assessed to be around 44.7 and 59.5%, respectively [13]. In 2012, the World Health Assembly supported a 50% decrease in the prevalence of anemia in women who are fertile [9, 14]. The Ethiopian government has been working hard to reduce the high prevalence of micronutrient deficiencies, such as anemia, by implementing national programs and strategies like the National Nutrition Program (NNP) and the micronutrient deficiency control strategy [13], but this sustained effort has not yet been sufficient to meet both regional and national-level targets. Evidence from various sources indicates that among the sociodemographic, economic, and environmental risk factors for anemia are residence [3, 15, 16], low maternal education, poor wealth index [9, 16], household size, interpregnancy interval [7, 15], being currently pregnant, toilet facilities, being HIV positive [16], and parity [17]. Anemia still has nearly double (or twice) the burden problem in the regions of Afar and Somalia, despite Ethiopia's efforts to reduce the burden through improved coverage, crucial antenatal care follow-ups, and monitoring of health problems and their factors, which are essential for developing effective interventions [13]. On the prevalence and related elements of current, updated information in the research settings, there was scant evidence. The authors, taking into account numerous sociodemographic, economic, and environmental determinants in the study settings, undertook an exhaustive cross-sectional analysis of the most current 2016 EDHS report to identify the primary risk factors for anemia in the two regions and fill this gap [13]. Therefore, the primary goal of this study was to evaluate the prevalence of anemia in women in the two extremely anemic districts of Ethiopia and to identify associated risk factors.

## 2. Methods

### 2.1. Study Design, Period, and Data Source

From January 18, 2016 to June 27, 2016, a retrospective community-based cross-sectional study design utilizing secondary data analysis of the 2016 EDHS report dataset was used nationwide.

By visiting the official DHS program database at https://www.DHSprogram.com and outlining the main goal of our study in an online request, authorization was obtained to acquire this data set from the 2016 EDHS report.

### 2.2. Sampling Procedure and Study Population

**Figure 1:**
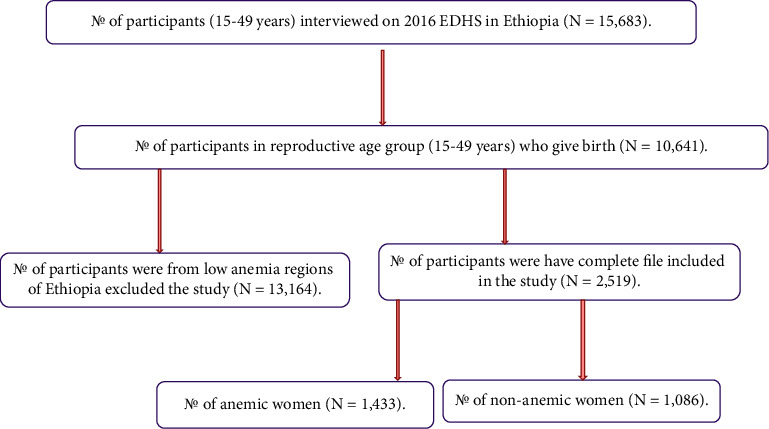
Schematic representation of the sampling procedure among women in two highly anemic regions of Ethiopia.

The stratified random sample method and staged selection were both employed in the 2016 EDHS report. First, 645 enumeration locations were chosen, 443 of which were in rural areas and 202 in urban ones. Second, from the newly formed household lists, a set number of 28 households per strata were chosen using an equal probability systematic random selection approach. The comprehensive 2016 EDHS report [13] included a display of the stratified random sampling method in detail. The 2016 EDHS survey indicated that the Afar and Somali areas had the highest anemia rates; hence, these regions were both chosen. The study included every woman in the reproductive age range (15–49 years old). In total, 2,519 women between the ages of 15–49, who were fertile participated in this study. Women with low levels of anemia within the time period specified were not included in the study (Figure 1).

### 2.3. Ordinal Logistic Regression Model

When the response variables contain multiple (polytomous) categories, this happens frequently. Response variables can be divided into ordinal and multinomial groups. The multinomial logistic regression model analysis cannot be used when the response variable is categorized in a certain order of categories. In these situations, ordinal outcome variables have been analyzed using an ordinal logistic regression model. A natural option for ordinal datasets is multivariable analysis when it is necessary to take into account many risk factors. The ordinal logistic regression model is one of the most widely used regression model types in logistic analysis. The constrained cumulative logit model is the ordinal logistic regression analysis that is most frequently useful [18, 19].

### 2.4. The Cumulative Logit Model

When the response variable has an order, ordinal logistic regression analysis is used. The proportional odds model, commonly referred to as the cumulative probabilities of the outcome categories, is the most commonly used ordinal logistic regression model. The use of ordinal logistic analysis is the recommended method if we suppose that the response variable is recorded as ordinal with polytomous categories. Modeling the cumulative logit has frequently been used in an evaluation to expand the logistic regression analysis for dichotomous responses to accommodate polytomous ordinal response variables. Because the levels of anemia in women are ordered, ordinal logistic regression analysis was performed in this study. As a result, the proportional odds logistic regression model was applied to the categorical variable *Y* with *C* ordered categories in order to evaluate the risk factors of anemia degree, and a collection of *P* predictor variables for the *j*^th^ subject *X*′*j* = (*x*_1*j*_, *x*_2*j*_, *x*_3*j*_ …, *x*_*pj*_), *j* = 1, 2, 3,…, *n* is presented as follows:

(1) $$\begin{array}{c} \text{Logit}\left[Y_{\left.j\leq i\right|xj}\right]=\log\left[\frac{\pi_{i}\left(X_{j}\right)}{1-\pi_{i}\left(X_{j}\right)}\right]\\ =\alpha_{i}-\beta_{1}x_{1j}+{\beta_{2}x}_{2j}\ldots +{\beta_{p}x}_{pj}, \end{array}$$

where *π*_*i*_(*X*_*j*_) = is the probability of anemia level for women (pr(Y_*j* ≤ *i*|*xj*_)), *i* = 1, 2, 3,…, *c* − 1, *j* = 1, 2, 3,…, *n*, *β* = is a column vector of *P* regression coefficients, *α*_*i*_ = is *i*^th^ intercept coefficient, and *x*_*p*_ = is the number of predictors.

The test of the parallelism assumption was assessed once the best model analysis had been selected. A nonsignificant chi-square test of the parallelism assumption indicates that the logit surfaces are parallel and that the odds ratios can be interpreted as constants across all possible cut-off points of the response variable.

### 2.5. Study Variables

The anemia level served as the response variable. It is an ordered categorical variable (i.e., nonanemic, mild, moderate, and severe anemia). According to WHO guidelines [13, 20], the severity of anemia in women was divided into three categories:

1. Mild anemia: Hgb = 10.0–11.9 g/dl
2. Moderate anemia: Hgb = 7.0–9.9 g/dl
3. Severe anemia: Hgb < 7.0 g/dl

Table 1 includes a full definition and categorization of the predictor factors that were found after researching the prior literature.

### 2.6. Data Management and Analysis

Software STATA/SE version 12 was used to examine the encoded data after the data had been cleaned, decoded, and extracted using SPSS version 20. Tables and figures were used to present the data in a descriptive manner, describing the study participants. A Hosmer–Lemeshow (Pearson chi-square test) goodness-of-fitness test was used to gauge the model's fitness.

Analyses using bivariable and multivariable ordinal logistic regression models were carried out. The multivariable analysis took into account each variable from the bivariable analyses. Finally, the strength of the link was evaluated using the adjusted odds ratio (AOR) and its 95% confidence interval (CI), and variables with a *p* value of 0.05 or less were deemed statistically significant risk factors for anemia.

## 3. Results

### 3.1. Sociodemographic Characteristics of the Study Participants

A total of 2,519 women participants were included in the study. Of them, respectively, 47.85, 29.4, 19.35, and 3.4% were not anemic, mildly, moderately, or severely. Women who lived in rural regions had anemia in varying degrees (27.8% had mild anemia, 26.9% had moderate anemia, and 3.4% had severe anemia). For women who lived in urban areas, these percentages were 15.3, 9.2, and 1.0%, respectively. Anemia was more prevalent at mild, moderate, and severe levels in families without improved toilet facilities (28.6, 28.5, and 4.7%, respectively) than it was in households with standard toilet facilities (Table 2).

### 3.2. Chi-Square Test of Association

Greater than in the Afar area, anemia was more severe in the Somali regional state of 64 (4.6%). Regional variation and anemia level were significantly correlated (*p* value ≤0.000). Anemia was more common in women who were positive (pregnant) than in those who were negative (not pregnant). Additionally, a strong correlation between anemia level and the mother's current diarrheal condition has been found (*p* value = 0.002).

According to the chi-square test, the presence of anemia is related to region, birth type, diarrheal status, religion, wealth index, use of contraceptives, being currently pregnant, residence, toilet facility, ANC follow-up, and marital status (*P* < 0.05) (Table 2).

### 3.3. Prevalence of Anemia among Women

**Figure 2:**
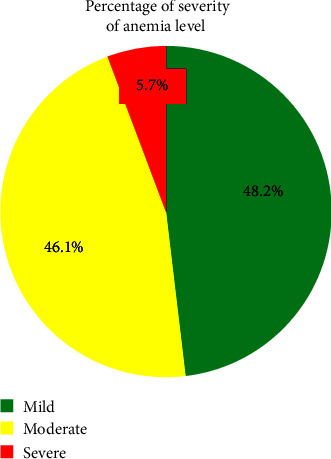
Severity of anemia level among women in two highly anemic regions of Ethiopia, January 18, 2016, to June 27, 2016.

Anemia was found to be prevalent overall in this study at 56.8% (95% CI (54.8%−58.7%)). 48.2% of women had mild anemia, 46.1% had moderate anemia, and 5.7% had severe anemia, according to the degree of anemia (Figure 2).

### 3.4. Ordinal Logistic Regression

#### 3.4.1. Factors Associated with Anemia among Women

According to the results of the bivariable ordinal logistic regression model analysis, the variables husband's educational level, family size, marital status, and child's gender were not significant at the 5% level of significance. Region, usage of contraceptives, parity, being currently pregnant, ANC follow-up, toilet facility, and marital status were all found to be significant predictors of anemia in the multivariable ordinal logistic regression model analysis.

The Pearson-based chi-square test for this model analysis produced a chi-square value of 4555.660 (*p* value = 0.524), indicating that the model and its dataset had a better fit. The chi-square test of the parallelism assumption also showed that the odds ratios of the final model remained constant across all cut-off values for women's anemia levels, with a chi-square value of 61.8 (*p* value = 0.122) at the 5% level of significance.

According to Table 3, those who used contraception had a 3.0 (AOR = 3, 95% CI: 2.5, 3.6) times greater chance of experiencing a higher anemia level than those who did not use contraception. Women in the Somali area had a 1.6 (AOR = 1.6, 95% CI: 1.37, 1.90) times higher risk of getting anemia compared to women living in the Afar regional state.

Accordingly, women who were already pregnant had a 2.8 (AOR = 2.8, 95% CI: 2.3, 3.4) times higher risk of developing a higher anemia level than nonpregnant women.

In comparison to women who had 0–3 total ever-born children, those who had 4-5 total ever-born children were 1.3 (AOR = 1.3, 95% CI: 1.05–1.66) times more likely to experience anemia. Similar to this, women with 6 or more total children born in their lifetimes had a 1.4 (AOR = 1.4, 95% CI: 1.1, 1.7) times higher risk of developing anemia than women with 0 to 3 total children.

In addition, the findings demonstrated that women who did not attend ANC had a higher likelihood of acquiring a higher anemia level (AOR = 1.9, 95% CI: 1.6, 2.3). On the other hand, the availability of unimproved toilet facilities had a positive impact on developing anemia levels. Women with unimproved toilet facilities had a 30% higher risk of having anemia than women with improved toilet facilities (AOR = 1.3, 95% CI: 1.1, 1.6). Finally, it was discovered that the women's marital status had a substantial impact on how anemic they were. In comparison to women who had never married, married women had a 1.4 (AOR = 1.4, 95% CI: 1.1, 1.9) times higher risk of developing anemia.

## 4. Discussion

Anemia is a worldwide problem that affects all individual groups. The two populations that are most susceptible to anemia are women and children. The prevalence of anemia among women overall in the current study was 56.8% (95% CI (54.8%−58.7%)).

This result is consistent with research done in Nigeria, 54.5% [21], Ghana, 57.1% [22], Ethiopia, 52% [23], and 53% [24]. However, this finding is higher than another study done by the Ethiopian DHS in 2005, 27.4%, and the EDHS in 2016, 24% [13], and in Southeast Ethiopia, 27.9% [25, 26] and 27.6% [15]. This study, however, falls short of those carried out in Burkina Faso, 61% [27], and Uganda, 63.1% [28]. Geographical, socioeconomic, seasonal, nutritional, and behavioral variables may all play a role in the disparity [11, 29]. Another explanation can be related to the presence or absence of intestinal parasite infections and malaria, which are risk factors that also alter the degree of anemia [30].

Multivariable ordinal logistic regression analysis revealed a significant risk factor relationship between anemia level and marital status, parity, toilet facility, usage of contraceptives, and being currently pregnant.

Women who live in the Somali region are 1.6 times more likely to have anemia than women who live in the Afar region (AOR = 1.6, 95% CI: 1.37, 1.90). The inaccessibility and unavailability of health care facilities may be the cause of the increased prevalence of anemia among women from the Somali region. As a result, they are ignorant of the risk factors contributing to anemia and the available preventative measures. Similar to this, pregnant women had higher anemia levels than nonpregnant women did. This result is in line with a study that was done in Ethiopia [16]. According to the current research, married women have 1.4 times higher odds of having anemia than unmarried or single women (AOR = 1.4, 95% CI: 1.1, 1.9). This may be because married women are more likely to become pregnant and have their first child.

Women who had not had an ANC follow-up had anemic probabilities that were 1.9 times higher than those who did. This outcome is in line with the Addis Ababa study's findings [15]. This discrepancy from other studies conducted in Ethiopia [29] could be because women who attended ANC follow-up received assistance from medical practitioners to ward off anemia. Importantly, the ANC provides counseling and follow-up support to encourage women to use iron-rich supplements and drugs that also contain folic acid. A statistically significant correlation between parity and the probability of developing anemia was found in the current investigation. According to study findings, women in Iran [17] and Pakistan [31, 32] with parities of four or higher were found to have a higher risk of getting anemia than women with lower parities. This explanation could be because women who give birth frequently have increased blood loss, which lowers hemoglobin levels in the body [33]. Another explanation is that women who have high parity are more likely to get anemia since it is typically difficult for families to provide enough food and medical treatment for all of their women. As a result, severe malnutrition and diseases may be more easily transmitted to women.

Contrary to their counterparts, women who had utilized contraceptive techniques had higher rates of anemia. In this study, the risk of developing anemia was three times higher in women who utilized contraceptive methods.

The results of this study also showed that women were more likely to be anemic in families with unimproved toilet facilities than in households with improved toilet facilities. This result is consistent with research conducted in Benin [2, 34]. The explanation might be that in Ghana, unimproved latrines exposed women to helminthic infections [35], which led to the development of anemia in them [36].

### 4.1. Limitations of the Study

The limitation of the study is that some significant predictor variables, such as the hookworm infection, gestational age of mothers, and dietary information, were left out of the study.

Another limitation of this study was the recall bias of the questionnaire-based survey, which relied on the memory of the women. Moreover, this investigation was done four years ago, so it is unlikely to reflect the latest status of the mortality rate in the pastoral region of Ethiopia.

## 5. Conclusions

As a result, the anemia burden is considerably greater than the national average. The region, usage of contraceptives, being pregnant at the time, ANC follow-up, toilet facilities, parity, and marital status all had a substantial impact on anemia. Therefore, to lessen the prevalence of anemia in certain parts of Ethiopia, public health initiatives that improve maternal health service consumption are required, such as ANC follow-up to minimize parity. Additionally, raising awareness of the dangers of anemia and offering helpful guidance in the other areas where gaps have been found should lessen the current burden of anemia among women in the research area.

## Figures and Tables

**Table 1:** Operational definitions and categorizations of predictor variables.

No	Variables	Categorizations of predictor variables
1	Region	Place of region type (Afar, Somali)
2	Birth type	Type of birth child (single, multiple)
3	Maternal education level	Mother's education level (tertiary, secondary, primary, no education)
4	Diarrheal status	Mother's diarrheal status (no, yes)
5	Religion	Type of religion (orthodox, muslim, others^*∗*^)
6	Family size	Numbers of household members (<6, ≥6)
7	Sex of household head	Sex of household head (male, female)
8	Wealth index	Wealth index of households (rich, middle, poor)
9	Contraceptive use	Mother's used contraceptive method (no, yes)
10	Sex of child	Sex of child (male, female)
11	Parity	Children ever born in household (0–3, 4-5, ≥6)
12	Currently pregnant	Mother's currently pregnant status (no, yes)
13	Residence	Place of residence (urban, rural)
14	Toilet facility	Mother's used improved type of toilet facility (yes, no)
15	ANC follow up	Mother's ANC follow up during pregnancy (yes, no)
16	Marital status	Mother's marital status (single, married, others^*∗∗*^)
17	Husband education level	Mother's husband education level (tertiary, secondary, primary, no education)

Others^*∗*^: protestant/catholic/pagan, others^*∗∗*^: divorced/widowed/separate.

**Table 2:** Percentage distribution and test of association between anemia level and predictor variables (*N* = 2,519).

Variables	Categories	Chi-square analysis					
		Mother's anemia level					
		Nonanemic *N*(%)	Mild *N*(%)	Moderate *N*(%)	Severe *N*(%)	*x* ^2^	*P* value
Region	Afar	562 (49.8%)	311 (27.6%)	237 (21%)	18 (1.6%)	59.7	≤0.000^*∗*^
	Somali	525 (37.7%)	378 (27.2%)	424 (30.5%)	64 (4.6%)		

Birth type	Single	284 (53.50%)	125 (23.5%)	118 (22.2%)	4 (0.80%)	37.3	≤0.000^*∗*^
	Multiple	803 (40.4%)	564 (28.4%)	543 (27.3%)	78 (3.9%)		

Maternal education level	Tertiary	32 (58.2%)	8 (14.5%)	15 (27.3%)	0 (0.00%)	37.5	≤0.000^*∗*^
	Secondary	62 (54.9%)	28 (24.8%)	22 (19.5%)	1 (0.90%)		
	Primary	244 (49.9%)	132 (27.0%)	105 (21.5%)	8 (1.6%)		
	No education	749 (40.2%)	521 (28.0%)	519 (27.9%)	73 (3.9%)		

Diarrheal status	No	340 (48.6%)	186 (26.6%)	159 (22.7%)	15 (2.1%)	15.1	0.002^*∗*^
	Yes	747 (41.1%)	503 (27.7%)	502 (27.6%)	67 (3.7%)		

Religion	Orthodox	65 (73.0%)	12 (13.5%)	12 (13.5%)	0 (0.00%)	37.7	≤0.000^*∗*^
	Muslim	1006 (42%)	670 (27.9%)	644 (26.8%)	82 (3.4%)		
	Others^*∗*^	16 (57.1%)	7 (25.0%)	5 (17.9%)	0 (0.00%)		

Family size	<6	533 (44.5%)	309 (25.8%)	312 (26.1%)	43 (3.6%)	3.8	0.284
	≥6	554 (41.9%)	380 (28.7%)	349 (26.4%)	39 (3.0%)		

Sex of household head	Male	614 (41.7%)	399 (27.1%)	403 (27.4%)	55 (3.7%)	6.0	0.110
	Female	473 (45.1%)	290 (27.7%)	258 (24.6%)	27 (2.6%)		

Wealth index	Rich	42 (40.8%)	23 (22.3%)	32 (31.1%)	6 (5.80%)	56.7	≤0.000^*∗*^
	Middle	301 (56.1%)	122 (22.7%)	110 (20.5%)	4 (0.70%)		
	Poor	744 (39.6%)	544 (29.0%)	519 (27.6%)	72 (3.8%)		

Contraceptive use	No	824 (53.3%)	352 (22.8%)	328 (21.2%)	41 (2.7%)	169	≤0.000∗
	Yes	263 (27.0%)	337 (34.6%)	333 (34.2%)	41 (4.2%)		

Sex of child	Male	410 (42.1%)	276 (28.4%)	253 (26.0%)	34 (3.5%)	1.29	0.732
	Female	677 (43.8%)	413 (26.7%)	408 (26.4%)	48 (3.1%)		

Parity	0–3	227 (44.7%)	152 (29.9%)	118 (23.2%)	11 (2.2%)	11.6	0.072
	4-5	235 (39.4%)	170 (28.5%)	165 (27.7%)	26 (4.4%)		
	≥6	625 (44.2%)	367 (25.9%)	378 (26.7%)	45 (3.2%)		

Currently pregnant	No	976 (51.5%)	486 (25.7%)	398 (21.0%)	34 (1.8%)	262	≤0.000∗
	Yes	111 (17.8%)	203 (32.5%)	263 (42.1%)	48 (7.7%)		
Residence	Urban	73 (74.5%)	15 (15.3%)	9 (9.2%)	1 (1.00%)	41.5	≤0.000^*∗*^
	Rural	1014 (41.9%)	674 (27.8%)	652 (26.9%)	81 (3.4%)		

Toilet facility	Yes	619 (47.8%)	339 (26.2%)	312 (24.1%)	25 (1.9%)	33.8	≤0.000^*∗*^
	No	468 (38.2%)	350 (28.6%)	349 (28.5%)	57 (4.7%)		

ANC follow-up	Yes	785 (52.4%)	347 (23.1%)	334 (22.3%)	33 (2.2%)	131	≤0.000^*∗*^
	No	302 (29.6%)	342 (33.5%)	327 (32.1%)	49 (4.8%)		

Marital status	Single	217 (46.4%)	114 (24.4%)	129 (27.6%)	8 (1.70%)	13.5	0.036^*∗*^
	Married	767 (41.6%)	522 (28.3%)	486 (26.4%)	69 (3.7%)		
	Others^*∗∗*^	103 (49.8%)	53 (25.6%)	46 (22.2%)	5 (2.40%)		

Husband education level	Tertiary	41 (44.1%)	21 (22.6%)	28 (30.1%)	3 (3.20%)	10.3	0.323
	Secondary	67 (51.5%)	31 (23.8%)	26 (20.0%)	6 (4.60%)		
	Primary	122 (47.5%)	69 (26.8%)	58 (22.6%)	8 (3.10%)		
	No education	857 (42.0%)	568 (27.9%)	549 (26.9%)	65 (3.2%)		

Others^*∗*^: protestant/catholic/pagan, others^*∗∗*^: divorced/widowed/separate, *x*^2^: chi-square.

**Table 3:** Bi-variable and multivariable analysis of ordinal logistic regression for factors associated with anemia among women in two highly anemic regions of Ethiopia (*N* = 2,519).

Variables	Categories	Ordinal logistic regression result	
		Bi-variable analysis	Multivariable analysis
		COR (95% CI)	AOR (95% CI)
Region	Afar (ref.)		
	Somali	1.7 (1.5, 2.0)^*∗*^	1.6 (1.37, 1.90)^*∗*^

Birth type	Single (ref.)		
	Multiple	1.66 (1.38, 1.99)^*∗*^	0.92 (0.68, 1.20)

Maternal education level	Tertiary (ref.)		
	Secondary	0.99 (0.5, 1.94)	0.81 (0.44, 1.5)
	Primary	1.2 (0.66, 2.2)	0.96 (0.55, 1.7)
	No education	1.8 (1.0, 3.2)^*∗*^	1.3 (0.74, 2.3)

Diarrheal status	No (ref.)		
	Yes	1.37 (1.2, 1.6)^*∗*^	1.1 (0.87, 1.3)

Religion	Orthodox (ref.)		
	Muslim	3.6 (2.2, 5.90)^*∗*^	1.3 (0.76, 2.2)
	Others^*∗*^	1.9 (0.82, 4.4)	0.85 (0.4, 1.8)

Family size	<6 (ref.)		
	≥6	1.05 (0.91, 1.20)	1 (0.84, 1.2)

Sex of household head	Male (ref.)		
	Female	0.84 (0.73, 0.98)^*∗*^	0.93 (0.79, 1.1)

Wealth index	Rich (ref.)		
	Middle	0.47 (0.31, 0.73)^*∗*^	0.66 (0.4, 1.1)
	Poor	0.9 (0.60, 1.34)	0.83 (0.51, 1.34)

Contraceptive use	No (ref.)		
	Yes	2.5 (2.14, 2.9)^*∗*^	3 (2.5, 3.6)^*∗*^

Sex of child	Male (ref.)		
	Female	0.96 (0.83, 1.1)	1 (0.84, 1.2)

Parity	0–3 (ref.)		
	4-5	1.3 (1.06, 1.60)^*∗*^	1.3 (1.05, 1.66)^*∗*^
	≥6	1.1 (0.92, 1.30)	1.4 (1.1, 1.7)^*∗*^

Currently pregnant	No (ref.)		
	Yes	4 (3.30, 4.60)^*∗*^	2.8 (2.3, 3.4)^*∗*^

Residence	Urban (ref.)		
	Rural	4 (2.5, 630)^*∗*^	1.5 (0.938, 2.5)

ANC follow-up	Yes (ref.)		
	No	2.2 (1.90, 2.60)^*∗*^	1.9 (1.6, 2.3)^*∗*^

Toilet facility	Yes (ref.)		
	No	1.5 (1.27, 1.70)^*∗*^	1.3 (1.1, 1.6)^*∗*^

Marital status	Single (ref.)		
	Married	1.16 (0.96, 1.41)	1.4 (1.1, 1.9)^*∗*^
	Others^*∗∗*^	0.85 (0.62, 1.16)	1.3 (0.92, 1.9)

Husband education level	Tertiary (ref.)		
	Secondary	0.71 (0.42, 1.21)	0.84 (0.47, 1.5)
	Primary	0.8 (0.503, 1.26)	0.69 (0.42, 1.14)
	No education	0.99 (0.67, 1.50)	0.87 (0.55, 1.4)

/cut1 (ref.)			
/cut1	—		2.0 (0.89, 3.05)
/cut2	—		3.3 (2.3, 4.4)^*∗*^
/cut3	—		6.1 (5, 7.2)^*∗*^

*Note.* LRT (*X* ^ 2 (DF = 25)) = 582.463 and *P* < 0.05, Hosmer–Lemeshow test [*X*^2 (DF = 4562)] = 4555.660 and *p* value = 0.524, test of parallel lines (*X* ^ 2 (DF = 50)) = 61.805 and *p* value = 0.122, ref.: reference, ^*∗*^significance at *p* value <0.05, others^*∗*^: protestant/catholic/pagan, others^*∗∗*^: divorced/widowed/separate.

## Data Availability

Data for this study were sourced from Demographic and Health surveys (DHS), which is freely available online at https://dhsprogram.com.

## Abbreviations

ANC: Antenatal care
AOR: Adjusted odds ratio
CI: Confidence intervals
Hgb: Hemoglobin
COR: Crude odds ratio
EDHS: Ethiopian demographic and health survey
NNP: National nutrition program
LRT: Likelihood ratio test
SPSS: Statistical package for social science
SSA: Sub-Saharan Africa
WHO: World Health Organization.

## References

[1] Gebreweld A., Ali N., Ali R., Fisha T. (2019). Prevalence of anemia and its associated factors among children under five years of age attending at Guguftu health center, South Wollo, Northeast Ethiopia. *PLoS One*.

[2] Harding K. L., Aguayo V. M., Namirembe G., Webb P. (2018). Determinants of anemia among women and children in Nepal and Pakistan: an analysis of recent national survey data. *Maternal and Child Nutrition*.

[3] Berhe B., Mardu F., Legese H. (2019). Prevalence of anemia and associated factors among pregnant women in Adigrat General Hospital, Tigrai, northern Ethiopia, 2018. *BMC Research Notes*.

[4] Alemayehu M., Meskele M., Alemayehu B., Yakob B. (2019). Prevalence and correlates of anemia among children aged 6-23 months in Wolaita Zone, Southern Ethiopia. *PLoS One*.

[5] Muchie K. F. (2016). Determinants of severity levels of anemia among children aged 6–59 months in Ethiopia: further analysis of the 2011 Ethiopian demographic and health survey. *BMC Nutrition*.

[6] Afeworki R., Smits J., Tolboom J., van der Ven A. (2015). Positive effect of large birth intervals on early childhood hemoglobin levels in Africa is limited to girls: cross-sectional DHS study. *PLoS One*.

[7] Abay A., Yalew H. W., Tariku A., Gebeye E. (2017). Determinants of prenatal anemia in Ethiopia. *Archives of Public Health*.

[8] Carneiro I. A., Smith T., Drakeley C. J., Lusingu J. P. A., Malima R., Utzinger J. (2006). Modeling the relationship between the population prevalence of Plasmodium falciparum malaria and anemia. *The American Journal of Tropical Medicine and Hygiene*.

[9] Ejigu B. A., Wencheko E., Berhane K. (2018). Spatial pattern and determinants of anaemia in Ethiopia. *PLoS One*.

[10] Melku M., Addis Z., Alem M., Enawgaw B. (2014). Prevalence and predictors of maternal anemia during pregnancy in Gondar, Northwest Ethiopia: an institutional based cross-sectional study. *Anemia*.

[11] Addis Alene K., Mohamed Dohe A. (2014). Prevalence of anemia and associated factors among pregnant women in an urban area of Eastern Ethiopia. *Anemia*.

[12] Kassebaum N. J., Jasrasaria R., Naghavi M. (2014). A systematic analysis of global anemia burden from 1990 to 2010. *Blood*.

[13] Central statistical agency (CSA) [Ethiopia] and ICF (2016). *Ethiopia Demographic and Health Survey 2016. Addis Ababa, Ethiopia, and Rockville*.

[14] Gonete K. A., Tariku A., Wami S. D., Derso T. (2018). Prevalence and associated factors of anemia among adolescent girls attending high schools in Dembia District, Northwest Ethiopia, 2017. *Archives of Public Health*.

[15] Getahun W., Belachew T., Wolide A. D. (2017). Burden and associated factors of anemia among pregnant women attending antenatal care in southern Ethiopia: cross sectional study. *BMC Research Notes*.

[16] Kibret K. T., Chojenta C., D’Arcy E., Loxton D. (2019). Spatial distribution and determinant factors of anaemia among women of reproductive age in Ethiopia: a multilevel and spatial analysis. *BMJ Open*.

[17] Sadeghian M., Fatourechi A., Lesanpezeshki M., Ahmadnezhad E. (2013). Prevalence of anemia and correlated factors in the reproductive age women in rural areas of tabas. *Journal of Family and Reproductive Health*.

[18] McCullagh P. (1980). Regression models for ordinal data. *Journal of the Royal Statistical Society: Series B*.

[19] Hosmer D. W., Lemeshow S., Cook E. (2000). *Applied Logistic Regression*.

[20] De Benoist B., Cogswell M., Egli I., McLean E. (2008). *Worldwide Prevalence of Anaemia 1993-2005; WHO Global Database of Anaemia*.

[21] Olatunbosun O. A., Abasiattai A. M., Bassey E. A., James R. S., Ibanga G., Morgan A. (2014). Prevalence of anaemia among pregnant women at booking in the university of uyo teaching hospital, uyo, Nigeria. *BioMed Research International*.

[22] Glover-Amengor M., Owusu W. B., Akanmori B. D. (2005). Determinants of anaemia in pregnancy in sekyere west district, Ghana. *Ghana Medical Journal*.

[23] Mihiretie H., Anane Mitiku M. F., Mitiku A. (2015). Magnitude of anemia and associated factors among pregnant women attending antenatal care in Nekemte health center, Nekemte, Ethiopia. *Journal of Medical Microbiology & Diagnosis*.

[24] Getachew M., Yewhalaw D., Tafess K., Getachew Y., Zeynudin A. (2012). Anaemia and associated risk factors among pregnant women in Gilgel Gibe dam area, Southwest Ethiopia. *Parasites & Vectors*.

[25] Gebremedhin S., Enquselassie F. (2011). Correlates of anemia among women of reproductive age in Ethiopia: evidence from Ethiopian DHS 2005. *The Ethiopian Journal of Health Development*.

[26] Kefiyalew F., Zemene E., Asres Y., Gedefaw L. (2014). Anemia among pregnant women in Southeast Ethiopia: prevalence, severity and associated risk factors. *BMC Research Notes*.

[27] Douamba Z., Bisseye C., Djigma F. W. (2012). Asymptomatic malaria correlates with anaemia in pregnant women at Ouagadougou, Burkina Faso. *Journal of Biomedicine and Biotechnology*.

[28] Mbule A. M., Byaruhanga Y. B., Kabahenda M., Lubowa A. (2013). Determinants of anaemia among pregnant women in rural Uganda. *Rural and Remote Health*.

[29] Jufar A. H., Zewde T. (2014). Prevalence of anemia among pregnant women attending antenatal care at tikur anbessa specialized hospital, Addis Ababa Ethiopia. *Journal of Hematology and Thromboembolic Diseases*.

[30] Desalegn A., Mossie A., Gedefaw L. (2014). Nutritional iron deficiency anemia: magnitude and its predictors among school age children, southwest Ethiopia: a community based cross-sectional study. *PloS one*.

[31] Soofi S., Khan G. N., Sadiq K. (2017). Prevalence and possible factors associated with anaemia, and vitamin B_12_and folate deficiencies in women of reproductive age in Pakistan: analysis of national-level secondary survey data. *BMJ open*.

[32] Habib M. A., Raynes-Greenow C., Soofi S. B. (2018). Prevalence and determinants of iron deficiency anemia among non-pregnant women of reproductive age in Pakistan. *Asia Pacific journal of clinical nutrition*.

[33] Bh R., Patil P., Joseph J. (2017). Multigravidity a major risk factor of anaemia in pregnancy and its comparison in primigravida women in raichur. *National Journal of Laboratory Medicine*.

[34] Alaofè H., Burney J., Naylor R., Taren D. (2017). Prevalence of anaemia, deficiencies of iron and vitamin A and their determinants in rural women and young children: a cross-sectional study in Kalalé district of northern Benin. *Public health nutrition*.

[35] Mara D., Lane J., Scott B., Trouba D. (2010). Sanitation and health. *PLoS medicine*.

[36] Tay S. C. K., Nani E. A., Walana W. (2017). Parasitic infections and maternal anaemia among expectant mothers in the Dangme East District of Ghana. *BMC research notes*.

